# Lysyl Oxidase, A Critical Intra- and Extra-Cellular Target in the Lung for Cigarette Smoke Pathogenesis

**DOI:** 10.3390/ijerph8010161

**Published:** 2011-01-19

**Authors:** Wande Li, Jing Zhou, Lijun Chen, Zhijun Luo, Yinzhi Zhao

**Affiliations:** 1 Department of Biochemistry, Boston University School of Medicine, 72 East Concord Street, Boston, MA 02118, USA; E-Mails: jz@bu.edu (J.Z.); zluo@bu.edu (Z.L); yinzhi@bu.edu (Y.Z.); 2 Department of Pharmacology, Zhongshan Medical College, Sun Yat-Sen University, 74 Zhongshan Road II, Guangzhou, 510089, China; E-Mail: chenlij@mail.sysu.edu.cn (L.C.)

**Keywords:** cigarette smoke (CS), CS condensate (CSC), 4-(methylnitrosamino)-1- (3-pyridyl)-1-butanone (NNK), cadmium (Cd), lysyl oxidase (LO), emphysema, carcinogenesis

## Abstract

Cigarette smoke (CS), a complex chemical mixture, contains more than 4,800 different compounds, including oxidants, heavy metals, and carcinogens, that individually or in combination initiate or promote pathogenesis in the lung accounting for 82% of chronic obstructive pulmonary disease (COPD) deaths and 87% of lung cancer deaths. Lysyl oxidase (LO), a Cu-dependent enzyme, oxidizes peptidyl lysine residues in collagen, elastin and histone H1, essential for stabilization of the extracellular matrix and cell nucleus. Considerable evidences have shown that LO is a tumor suppressor as exemplified by inhibiting transforming activity of *ras*, a proto oncogene. CS condensate (CSC), 4-(methylnitrosamino)-1-(3-pyridyl)-1-butanone (NNK) and cadmium (Cd), major components of CS, down-regulate LO expression at such multiple levels as mRNA, protein and catalytic activity in lung cells *in vitro* and *in vivo* indicating LO as a critical intra- and extracellular target for CS pathogenesis in the lung. In view of multiple biological functions and regulation characteristics of the LO gene, molecular mechanisms for CS damage to lung LO and its role in emphysema and cancer pathogenesis are discussed in this review.

## 1. Introduction

Cigarette smoke (CS) is one of the most important risk factors for human disease, accounting for 82% of chronic obstructive pulmonary disease (COPD) deaths and 87% of lung cancer deaths [1–4]. Approximately 23% of the US adults currently smoke because of addiction to nicotine, an important component of CS. More than 440,000 Americans and approximately five million people in the World die prematurely each year as a result of tobacco use and tobacco smoke exposure [5]. Cigarettes kill more Americans than alcohol, car accidents, suicide, AIDS, homicide and illegal drugs combined [6].

Long-term exposure of humans to CS usually results in COPD in some people, often manifested as bronchitis, emphysema or both. Emphysema is a pathological lesion characterized by abnormal enlargement of the respiratory airspaces with destruction of the alveolar walls. Most studies of emphysema pathogenesis have focused on the “elastase-antielastase imbalance” hypothesis [7]. CS-activated macrophages and neutrophils release elastase that proteolyses elastin fibers contributing to lung injury [8,9]. However, this hypothesis cannot explain all aspects of the emphysema pathology. For example, the CS-induced emphysematous lung in guinea pigs exhibited breakdown of collagen without significant changes in elastin [10]. Transgenic mice that over-express the human collagenase gene developed early onset emphysema [11]. Thereby, the “elastase-antielastase imbalance” is not the only mechanism for emphysema pathogenesis.

Strong evidence supports the association of CS with lung cancer. CS is responsible for 772,000 and 265,000 new lung cancer cases in men and women, respectively, each year worldwide [12]. Passive smoke, also known as sidestream smoke (SS), environmental tobacco smoke (ETS) or second-hand smoke, may have more carcinogenic potentials than the mainstream smoke (MS) since it contains larger quantities of organic chemical compounds [13]. Cessation of smoking sharply declines pulmonary carcinogenesis in ex-smokers after approximately five years since quitting in comparison to continuous smokers [12], indicating CS as an important risk factor for lung cancers. Smoke-induced lung malignancies are mainly classified as squamous cell carcinoma, small cell carcinoma, adenocarcinoma, *etc.* [12]. Note that in addition to the lung, CS can also act as a critical cancer risk factor involved in other organs such as the larynx, bladder, esophagus, pancreas, kidney, oral cavity, *etc.* [12]. In light of its severe insults to the lung and other organs, CS is of a continuing human health concern. Thus, it is important and necessary to deeply investigate molecular mechanisms and preventing and therapeutic strategies for CS-induced lung diseases.

## 2. Major Pathogenic Agents of CS

### 2.1. CS, a Complex Chemical Mixture

CS contains more than 4,800 different compounds, including oxidants, heavy metals, and carcinogens, that individually or in combination initiate or promote pathogenesis in the lung [14,15]. Each puff of smoke produces 10^14^–10^16^ free radicals inducing oxidative damage to the lung [16]. The mainstream smoke coming from a burning cigarette is composed of a vapor phase (92%) and a particulate phase (8%). The major constituents of the vapor phase include carbon monoxide, ammonia, nitrogen oxides, hydrogen cyanide, isoprene, butadiene, formaldehyde, acetaldehyde, *etc.* The particulate phase or CS condensate (CSC) contains at least 3,500 compounds. There are 5 × 10^9^ particles/mL with diameters of 0.1–1 μm in the mainstream CS aerosol in comparison to 10^5^/mL aerosol particles with diameters 0.1–>10 μm in urban air. Thus, all of the particles in CS are within the size range reaching the alveolar space, known to be 0.1–3 μm [14]. Although the chemical composition of both MS and SS are quantitatively similar they still exhibit significant quantitative variety because of the temperature differences at which MS and SS are formed. Inhaled fresh sidestream cigarette smoke is approximately four times more toxic per gram total particulate matter (TPM) than mainstream cigarette smoke [17]. Based on our research focuses, two important classes of toxic/carcinogenic components in CSC such as the nicotine-derived nitrosamines, e.g., 4-(methylnitrosamino)-1-(3-pyridyl)-1-butanone (NNK), and heavy metals, e.g., cadmium (Cd) [14,15] will be discussed in this review.

### 2.2. NNK and Cd, Two of Major Pathogenic and Carcinogenic Components of CS

NNK, a tobacco-specific nicotine-nitrosated derivative, has been demonstrated as a very potent carcinogen in rodents, particularly in rats [18]. Its sales-weighted concentration in one current “full-flavored cigarette” is 131 ng [19]. The estimated life time exposure to NNK of a cigarette smoker is about 1.1 mg/kg. close to the lowest NNK dose (1.8 mg/kg) that induced tumors in rats [18,20]. NNK exhibits a remarkable affinity for the lung, resulting in adenoma and adenocarcinoma, independently of the route of administration [18]. Metabolic activation of NNK by cytochrome P-450 (CYP) is required to exert its carcinogenic activity [21]. Although it is established that NNK damages DNA via formation of methyl adducts [22,23] and mutation of p53, a tumor suppressor gene [24], the intracellular events elicited by CS/NNK and their underlying pathological mechanisms leading to cell injury and cancer development remain unclear.

Cd is a toxic metal without any biological availability. In addition to occupational exposure, cigarette smoke constitutes a major source of Cd exposure for humans since tobacco naturally absorbs mobile forms of Cd, e.g., CdCl_2_, CdSO_4_, Cd(NO_3_)_2_, Cd(CH_3_COO)_2_, *etc.*, from soil and accumulates relatively high concentrations of Cd in the leaves [25,26]. The lung, a major CS-target organ, not only absorbs but also accumulates Cd with a biological half-life of 9.4 years [25,27]. Each cigarette contains 1–2 μg Cd, of which 10% is inhaled [25,26]. Pulmonary Cd levels in smokers reached 7.5-fold greater than those in non-smokers [28]. Long-term exposure to Cd resulted in emphysema [29–31]. Fatal emphysema developed in Cd-poisoned patients who had survived acute lung injuries in industrial accidents [32]. Clinical correlation studies have established a causal relationship between smoking and human emphysema [33]. Smokers with severe emphysema have a high Cd levels in their lungs [25,28]. Animal studies indicated that the nature of Cd-induced lung lesions varied with the exposure protocols and species used. Both emphysema and fibrosis with and without airspace enlargement were produced in rodents [7,34]. Cd was listed by the US Environmental Protection Agency as one of 126 priority pollutants [35,36]. Cd is a potent human carcinogen and occupational exposure to Cd is associated with cancers in multiple organs such as the lung, the prostate, the pancreas, the kidney, *etc.* Because of its characteristics as a lung carcinogen, Cd was classified as a category 1 carcinogen by the International Agency for Research on Cancer (IARC) and the National Toxicology Program of the USA [25,36]. Animal studies provide strong evidence for Cd carcinogenesis. With the rodent model, mast relevant to human exposure to air-borne Cd, various Cd compounds produced adenocarcinomas in the lung of rats after inhalation [37,38]. Due to its negative results in bacterial mutagenic tests, Cd is predominantly considered as a non-genotoxic carcinogen [25], thus, the mechanism(s) of Cd carcinogenesis remain poorly understood.

Recent studies by this lab have shown that CSC down-regulates lysyl oxidase (LO), an intra- and extracellular enzyme with multiple biological functions, at mRNA, protein and catalytic levels [39–41]. This review intends to summarize our current findings indicating LO as a critical intra- and extracellular target for CS pathogenesis in the lung.

## 3. Lysyl Oxidase

### 3.1. Multiple Functions of LO in Biology

LO initiates the crosslinking of collagen and elastin by oxidizing specific peptidyl lysine residues in these proteins stabilizing collagen or elastin as fibers in the extracellular matrix (ECM) [41]. Thus, LO plays a central role in ECM morphogenesis and tissue repair. LO can also oxidize lysine residues in various globular proteins with basic isoelectric points (pI > 8), e.g., nuclear proteins histone H1 and H2 [42,43], bFGF [44], *etc.*, suggesting that electrostatic relationships between the anionic enzyme (pI ~ 6.0) and basic protein substrates are critical to substrate potential. In addition to extracellular secretion, LO is also localized within the nucleus [45] where it appears to modulate the packing state of nuclear chromatin [46]. Labeled 32 kDa mature LO readily enters the intracellular compartments and accumulates within the nucleus in vascular smooth muscle cells (VSMC) although mechanistic features of its intracellular transport into and through the cytosol are not known [47]. The 32 kDa purified LO displays chemotactic activities for monocytes and VSMC [48–50]. LO, as an extracellular signal, stimulates stress fiber formation and focal adhesion assembly [50] possibly implicating in mechanisms for tumor metastasis in hypoxia conditions [51]. The LO reaction produces H_2_O_2_ which may regulate gene expression and cell behavior [50]. LO is considered as a tumor suppressor gene initiating from the finding that expression of transfected LO cDNA suppressed Ha-*ras*-induced cell transformation indicating a *ras*-suppressor effect of LO [52]. Our previous studies have demonstrated bFGF as a substrate of LO. Oxidation of bFGF by LO blocked the proliferation of bFGF-stimulated normal cells and bFGF-autocrine transformed cells with high tumorigenic potential [44]. Based on the widening intra- and extracellular distribution of possible substrates of LO, this enzyme may display multiple functions in biology beyond its role in stabilization of macromolecules in the ECM.

### 3.2. LO Molecular Structure

**Figure 1:**
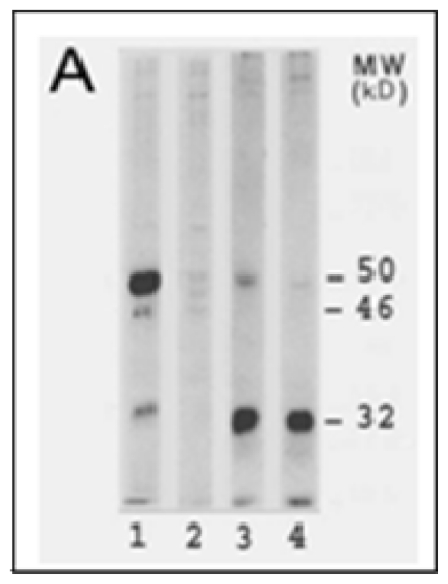

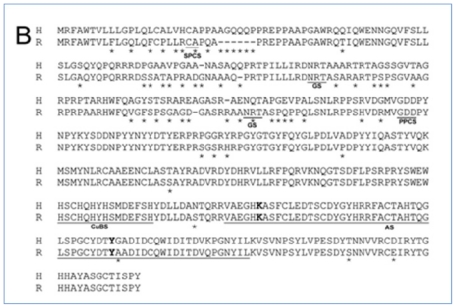
LO synthesis, processing and molecular composition. **A**, LO synthesis and processing. LO synthesis and processing were determined by [^35^S]-methionine labeling and cycloheximide chase (5 h) assays. 1 and 3, 0 time chase; 2 and 4, 5 h chase; 1 and 2, cell extracts; 3 and 4, conditioned media. Note that at 0 time chase the cell extract contains the 46 kDa prepro-LO, the 50 kDa pro-LO, and the membrane-bound 32 kDa mature LO while the conditioned medium contains the secreted 50 kDa pro-LO and the processed 32 kDa mature-LO; at 5 h chase all LO species in the cell extract were processed and secreted into the extracellular space, and further cleaved to the 32 kD mature enzyme. **B**, amino acid sequences of rat and human LO. SPCS, the signal peptide cleavage site; GS, the N-glycosylation site; PPCS, the propeptide cleavage site; AS, the active site; CuBS, the copper-binding site; asterisks indicate the different amino acid composition of LO between human and rat.

As shown in Figures 1A and 1B, LO is synthesized by fibrogenic cells as a 46 kD preproenzyme. Following signal peptide cleavage at the CA site (SPCS in Figure 1B) and N-glycosylation at the NRT site (GS in Figure 1B), the resulting 50 kD proenzyme is secreted and then proteolytically cleaved at the GDD site (PPCS in Figure 1B) to produce the 32 kD functional species in the extracellular space [41]. The sequences within the mature enzyme at which the active site (AS in Figure 1B) is located are highly conserved (95%) in rat and human (Figure 1B). LO specifically binds 1 g-atom of Cu(II) at its active site per mole of enzyme [41]. Five histidine residues located at the sequence of His 283–His 297 in rat LO (CuBS in Figure 1B) constitute the copper-binding motif. Cu binding to proLO occurs in the enzyme secreted pathway such as the Golgi apparatus [53]. In addition to Cu, LO also contains a covalently bound carbonyl cofactor identified as lysine tyrosylquinone (LTQ) deriving from Lys 314 and Tyr 349 (bold letter in Figure 1B) based on the sequence of rat LO [54]. This peptidyl orthoquinone functions as a transient electron sink during amine oxidation. The precise role of Cu in the LO action remains poorly understood. This metal ion may be essential for maintaining the structural integrity of LTQ and/or the protein [41].

The activity of purified LO can be severely inhibited by metal chelators, e.g., α,α′-dipyridyl [55]. The reaction with peptidyl lysines catalyzed by LO is: RCH_2_NH_2_ + H_2_O + O_2_ → RCHO + NH_3_ + H_2_O_2_ [41]. Additional LO-like cDNA species encode a variety of LO-like proteins, including LOXL, LOXL2, LOXL3 and LOXL4 [56] which have the homology at the C-terminal domain in comparison to the mature LO. Both LO and LO-like proteins at the C-terminal domain contain ten conserved cysteine residues, copper binding domains as well as tyrosine and lysine residues for forming the LTQ cofactor. Notably, LO also contains a growth factor and cytokine binding domain with the consensus: C-x_9–10_-C-x-W-x_26–32_-C-x_10–15_-C (C is Cys, W is Trp, x_n_ is a defined number of any amino acids) found in a superfamily of receptors for growth factors and cytokines evolved from fibronectin III sequence modules common in cell surface adhesion molecules [56–58].

### 3.3. LO Gene Core Promoter

In eukaryotic cells, the gene activation is regulated by the interaction of *cis*-elements with various protein factors at the gene promoter region. The RNA polymerase II (RNA-Poly II) complex binds to the core promoter initiating the gene transcription. Comparative LO gene sequence alignment across species of rat, human and mouse showed that the proximal promoter region is highly conserved whereas the distal promoter region was more divergent among three species suggesting a fundamental role of the proximal promoter region in the regulation of LO transcription. The maximal promoter activities were detected in the 804, 808 and 924 base pair regions, respectively, immediately upstream of ATG in rat, mouse and human LO genes [59]. Thus, the LO transcription control is likely to be regulated by similar elements in these species. The 5′-flanking region of the rat LO gene appears to contain no typical TATA box, the first core promoter element identified in RNA-Poly II-transcribed genes. Multiple transcription start sites cluster in the rat LO promoter region from −78 to −51 relative to ATG, one of which overlaps with the adenosine residue of the INR element (5′-TCATTTTT-3′) mapped from −53 to −46 in the rat LO promoter [60,61]. Furthermore, the sequence from −18 to −14, *i.e.*, 5′-GGACG-3′, a consensus sequence of the DPE that is located approximately 30 bp after the adenosine residue in the INR motif [61]. The INR and the DPE conserved in rat, human and mouse species coordinately function as a single core promoter unit for the RNA-Poly II-directed gene transcription [61]. It should be noted that the DPE in the rat LO gene promoter also worked as an independent core promoter module playing a key role in transactivation of the TATA-less LO gene at multiple transcription starts sites [59].

### 3.4. Redox-sensitive Cis-elements—the NFI-binding Site, HRE, MRE and ARE in the LO Promoter

**Figure 2:**
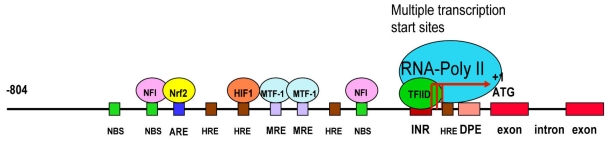
The schematic linear map of the cloned rat LO promoter. ATG, the translational start site; INR, the initiator element; DPE the down-stream core promoter element; NBS, the NFI binding site; HRE, the hypoxia response element; MRE, the metal response element; ARE, the antioxidant response element.

Other transcription factor binding sites (*cis*-acting elements) are generally located upstream of the core promoter and act through binding of different transcriptional activators and/or repressors to alter the level of gene transcription. In addition to putative binding sites for the following transcription factors such as nuclear factor I (NFI), SP1, Oct-1, C/EBPγ, *etc.*, the cloned rat LO promoter also contains the hypoxia response element (HRE), the metal response element (MRE) and antioxidant response element (ARE), *etc.* [59]. The cloned rat LO promoter −804/−1 yielding the maximal promoter activity contains three putative NFI-binding sites [59]. NFI binds to the consensus sequence TTGGC(N5)GCCAA (N = any nucleotides) on duplex DNA as a dimer. Notably, it can also bind to the individual half site, TTGGC or GCCAA, with a somewhat reduced affinity [62]. The highly conserved N-terminal residues of NFI contain the DNA binding domain whereas the proline-rich C-terminal residues constitute the transcriptional regulation domain [62]. Four cysteine residues are conserved in the DNA binding domain of all rat NFI isoforms sensitive to oxidative damage [63,64]. In addition, the rat LO promoter region −804/−1 also contains four putative hypoxia response elements (HRE, core sequence = RCGTG, R = purine) [65,66] including one on the coding strand and three on the noncoding strand at the region −457/−453, −387/−383, −194/−190 and −37/−33 (relative to ATG) [59], two putative metal response elements (MRE, core sequence = TGCRCNC, R = purine, N = any nucleotide) [67–69] located at −269/−263 and −248/−241, and one antioxidant response element (ARE, core sequence = RTGACNNNGC, R = purine, N = any nucleotides) [70] at the region −581/−572 [59]. The HIF1 is composed of HIF1α and HIF1β subunits [71]. The HIF1β is constitutively expressed whereas HIF1α is maintained at a low level in normoxic cells. Upon hypoxia, HIF1α is upregulated and HIF1 complexes bind to HREs transactivating hypoxia-inducible genes [65]. HIF1α has a unique domain sensitive to oxygen-dependant degradation. In normaxia, specific degradation of HIF1α is triggered through this domain by the proteasome [66]. Hypoxia is an important complication associated with lung diseases and tumors [72]. Reduced expression of HIF1α has been detected in the emphysema lung tissues in severe COPD patients [73], suggesting deregulation of HRE activities occurred in HIF1α targeting genes such as LO under this condition. Since cobalt chloride (CoCl_2_) stabilizes the HIF1α protein, this metal ion is used to mimic effects of hypoxia in cell study systems [74,75]. The MRE was initially found in multiple copies in metallothionein (MT) genes. MTs are cysteine-rich metal binding proteins essential for cellular metal metabolism and detoxification [68,69]. A protein that binds specifically to MREs is termed as MRE-binding transcription factor-1 (MTF-1) [67]. MTF-1 is a Zn finger transcription factor mediating the expression of MT genes which contain more copies of MREs. Cd and oxidative stress (such as H_2_O_2_) activate MTF-1 binding to the MT-I gene promoter by binding to or oxidation of other cellular protein sulfhydryls releasing bound Zn that in turn enhances MTF-1 affinity for DNA, thus activating MRE containing genes [67]. The transcriptional factor interacted with the ARE is the NF-E2-related factor-2 (Nrf2) which drives expressions of a variety xenobiotic metabolizing enzymes such as GST, NQO1, UGT, GCL, HO-1, GSH synthetase, γ-glutamyl transpeptidase, *etc.*, for antioxidant-detoxification [70]. Nrf2 is inactive in the cytoplasm by binding to the cysteine-rich Keap1 protein in the actin filaments. Upon oxidative stress, modification of cysteine residues in the Keap1 protein induces the Nrf2 release and nuclear translocation. After forming the heterodimer with Maf, Nrf2 binds to the ARE for transcriptional activation of the genes. CS exposure has been shown to generate reactive oxygen species (ROS) [16]. Transcription factors for the NFI binding site, HRE, MRE and ARE, are known sensitive to oxidative stress [62,66,69,70]. These redox-sensitive transcription factors will be addressed as potential targets for CS insult. A schematic linear map of the cloned rat LO promoter is shown in Figure 2.

## 4. LO as a Target of CS at Multiple Molecular Levels

### 4.1. Inactivation of LO Catalytic Activity by CSC and Cd

Previous studies by this lab have shown inhibition by CSC and Cd, major components of CS, of LO catalytic activity in treated cells [40,76,77]. LO is a metalloenzyme requiring Cu(II) as a cofactor for enzymatic function [41,55]. Dietary deprivation of Cu induced lathyritic injuries in animals due to suppression of LO activity [78–80]. It is known that thiol-containing MT is a critical modulator for Cu metabolism [81,82]. MT can chelate 11–12 moles of Cu/mole of protein [81] which are in the cuprous state [Cu(I)]. MT has a higher affinity for Cu than Cd since Cd selectively displaced Zn but not Cu in native calf liver MT, which binds four moles of Zn and three moles of Cu per mole of MT [83]. In addition to MT, glutathione (GSH) also functions in cellular Cu metabolism. GSH can form a complex with Cu-MT, providing the biological basis for Cu transfer between these two cellular thiols [82]. Reduction of Cu(II) to Cu(I) by GSH is thought to be a preliminary step in cellular transport of Cu. Cellular concentrations of GSH range from 0.1 to 10 mM, accounting for 90% of total cellular non-protein thiols. A rapid depletion followed by a later rebound increase of intra-cellular GSH was shown to occur in CS-treated lung cells and tissues consistent with marked increases of total GSH concentration in epithelial lining fluid (ELF) in cigarette smokers [84–87]. Our studies have illustrated CSC and Cd enhancement of GSH levels in cultured lung cells and in lung tissues of the animal model, and upregulation of the catalytic unit of γ-glutamylcysteine synthetase (GCS), a rate-limiting enzyme for GSH synthesis, at protein and mRNA levels indicating the activation status of GSH biosynthesis under CSC and Cd stress conditions [40,76,88]. Markedly elevated levels of MT and GSH were associated with considerably decreased catalytic levels of LO in CSC or Cd-treated RFL6 lung fibroblasts [40,76,77]. In light of the function of MT and GSH in Cu metabolism, CSC or Cd-induced elevation of MT and GSH in treated cells may largely trap cellular Cu, thus limiting its biological availability for LO leading to downregulation of LO activity that requires Cu as a cofactor [40,76,77]. Notably, isotope labeling assays have demonstrated that in MT-overexpressing RFL6 cells following long-term Cd exposure, radioactivity of ^64^Cu bound to the LO fraction amounted to only 9% of the control as compared to 1,400% of the control of ^64^Cu associated with the MT fraction [77]. Direct correlation of elevated levels of cellular thiols with downregulation of LO in CSC or Cd treated cells came from experiments in which RFL6 cells were exposed to GSH monoethyl ester (GME), a GSH delivery system [40], or transfected with MT cDNA [88]. Clearly, elevation of cellular GSH and MT by these molecular approaches was associated with reduction of LO activity in cell conditioned media. These results provide evidence directly linking perturbation of the homeostasis of cellular thiol and copper to the reduction of LO catalytic activity in model cells in response to CSC or Cd.

### 4.2. Inhibition of LO Protein Synthesis and Processing by CSC and Cd

CSC and Cd reduced levels of all LO protein species including the 46 kDa preproenzyme, the 50 kDa proenzyme and the 32 kDa mature enzyme in cultured lung fibroblasts [40,76,77]. This may result from upstream mechanisms such as decreases in LO mRNA. Notably, a stronger inhibition of downstream LO species, *i.e.*, the preproLO → the proLO → the mature LO, in cells treated with CSC under the same conditions, suggests that several steps in LO translational and posttranslational processing may be susceptible to CSC [40]. A major difference between Cd-resistant (CdR) RFL6 cells derived from long-term Cd exposure and parental RFL6 cells is the presence of the variant 52 kDa LO precursor in the former. The 52 kDa protein existed in the cell extract fraction but not the conditioned media, reflecting deficiency of secretion [76]. As revealed by double immunofluorescent staining, a number of large LO positive patches colocalized with the ER and nucleus structures in CdR cells, indicating an abnormal processing of LO precursor with its product accumulated in these organelles [76,77]. Since this variant LO precursor is apparently 2 kDa larger than the normal LO precursor, it may represent a molecular species without cleavage of the signal peptide, known to be a total of 21 amino acids in rat LO (Figure 1B). Another possibility to account for the variant LO proenzyme with a higher MW in CdR cells may be a different degree of N-glycosylation. It should be noted that the same 52 kDa protein recognized by the anti-LO antibody was also found in CdR-3T3 cells derived from Swiss 3T3 fibroblasts using the same Cd exposure regimen [89]. These results suggest that the variant proenzyme derived from abnormal LO processing at translational and posttranslational levels may represent an important characteristic of long term Cd exposure.

### 4.3. Downregulation of LO Transcription and Posttranscription by CSC/NNK and Cd

Both CSC and Cd decreased the steady-state level of LO mRNA in treated cells [39,40,76]. The steady-state concentrations of mRNA as determined by Northern blot are dependent on both the transcriptional rate and the mRNA stability. CSC treatment induced decreased LO transcriptional rates as determined by the nuclear run-on assay and enhanced LO mRNA instability as illustrated by the actinomycin D chase assay [39]. The data further indicated that lower doses of CSC (<40 μg/mL) were sufficient to perturb the LO mRNA stability whereas higher doses of CSC (>80 μg/mL) were required to interfere with its transcriptional initiation. Thus, inhibition of the transcriptional rate and the mRNA stability both collectively contributed to decreased levels of steady-state LO transcripts in cells exposed to CSC.

**Figure 3:**
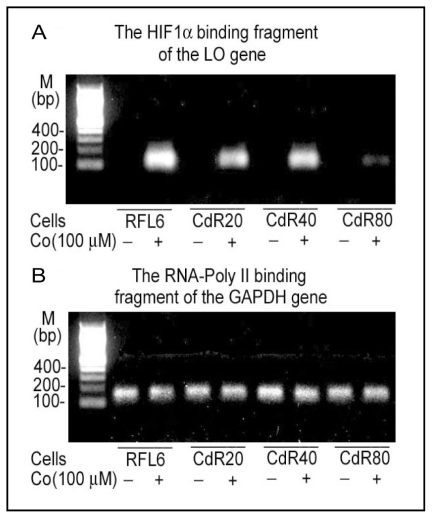
Cd effects on HIF1α binding to the LO promoter HRE. RFL6 cells and Cd-resistant (CdR) cells derived from RFL6 cells by long-term Cd exposure were treated without or with Co to mimic the hypoxia condition in the cell culture system. Cells resistant to 20, 40, and 80 μM Cd were referred to as CdR20, CdR40 and CdR80, respectively. HIF1α binding to the LO promoter HRE (−387/−383) in control and CdR cells were assessed by the ChIP assay (**A**) using RNA-Poly II binding to the GAPDH gene as an internal control (**B**) as described [59]. Primer pairs used in PCR are 5′-ctccctgtgcaacgtgtct-3′ [forward (F)] and 5′-tgcagttacacaagccgttc-3′ [Reverse (R)] for amplification of the HRE containing LO gene fragment (152 bp), and 5′-ttgcttggcttcttctttgg-3′ (F) and 5′-gagacgaggctggtactcca-3′ (R) for amplification of the GAPDH gene fragment (160 bp).

**Figure 4:**
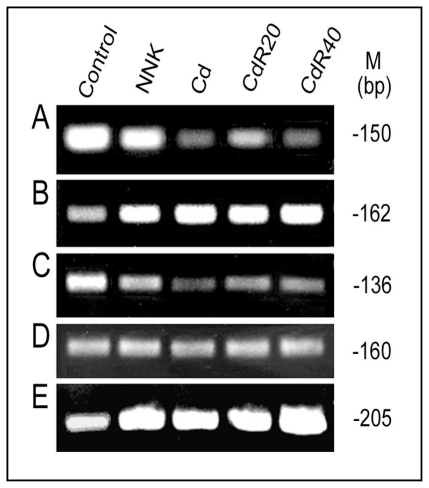
NNK and Cd effects on MTF-1, Nrf2 and acetylated histone H3 binding to and methylation of the LO promoter. MTF-1 binding to the LO MRE (**A**), Nrf2 binding to the LO ARE (**B**) and acetylated histone H3 binding to the LO core promoter region (**C**) in RFL6 control, NNK (300 μM for 48 h) and Cd (5 μM for 24 h) treated cells, and in CdR cells were assessed by the ChIP assays as described [59] using RNA-Poly II binding to the GAPDH gene as an internal control (**D**). Methylation of the LO core promoter region (**E**) was assessed by using the promoter methylation PCR kit (Panomics, Fremont, CA., USA). Primer pairs used in PCR are 5′-cttcagacactgtgcgctct-3′ (F) and 5′-gcagggactggtgccaag-3′ (R) for amplification of the MTF-1-bound LO MRE fragment (150 bp); 5′-tttggccctcatcgctct-3′ (F) and 5′-gacttaatctgggccgaaca-3′ (R) for amplification of the Nrf2-bound LO ARE fragment (162 bp); 5′-gaagaggtctccctccttcg-3′ (F) and 5′-actgcagctgtcccagaaag-3′ (R) for amplification of the acetylated histone H3-bound LO core promoter (INR-DPE) region (136 bp); 5′-ttgcttggcttcttctttgg-3′ (F) and 5′-gagacgaggctggtactcca-3′ (R) for amplification of the RNA Poly II-bound GAPDH gene fragment (160 bp); 5′-ttcagacactgtgcgctctc-3′ (F) and 5′-aggagggagacctcttcgag-3′ (R) for amplification of the methylated LO core promoter (INR-DPE) region (205 bp) containing 15 CpG islands.

To further investigate CSC modulation of LO transcription, an approximately 4 kb genomic fragment spanning nucleotides from −3,979 to −1 (Prom −3,979) was inserted in front of the luciferase gene in the reporter gene vector. A series of 5′-deletions of the Prom -−3,979 indicated that the maximal luciferase activity was found in the 804 base pair region immediately upstream of ATG [59]. CSC significantly inhibited LO promoter-directed luciferase expression in treated cells consistent with its effects on the LO transcriptional initiation [39]. Redox-sensitive transcription factors such as NFI, HIF1, MTF-1, *etc.*, are critical targets for CSC or Cd. CSC/NNK or Cd suppressed biological activities of the NFI binding sites, HRE, MRE except the ARE at the LO promoter region in interactions with corresponding transcription factors revealed by reporter gene activation, transcription factor cDNA expression and ChIP assays (Figures 3 and 4, and references [59,90,91]). At least two functional NFI binding sites (−594/−580 and −147/−133) are active in the 804 base pair promoter region of the LO gene. CSC inhibited NFI-binding site-directed luciferase activities in transfected cells in a dose-dependent manner and abolished the reporter gene activation by NFI A and B, two isoforms expressed in rat lung fibroblasts. The ChIP assays further confirmed CSC interferences with endogenous NFI incorporation with the LO gene in the RFL6 cell model [59,90]. Electrophoretic mobility shift, oligonucleotide competition and *in vitro* translation of HIF 1α assays indicated that only one HRE mapped at −387/−383 relative to the ATG was functionally active among 4 consensuses within the LO promoter region −804/−1 upstream of ATG. Cd enhanced cellular levels of reactive oxygen species (ROS) [92], thus inhibited HIF1α expression and binding to the LO gene in treated cells in the presence of CoCl_2_ (Co) that mimics hypoxia conditions in cell culture system revealed by ChIP and qPCR (Figure 3 and reference [91]). Cd significantly suppressed HIF1α mRNA expression in Cd-pulsed RFL6 cells and in Cd resistant cells stimulated by Co [91]. The cloned rat LO promoter (−804/−1 relative to ATG) also contains at least two MREs located at −269/−263 and −248/−241 and one ARE mapped at −581/−572. Unexpected, treatment of cells with Cd, a heavy metal, weakened but not enhanced MTF-1 binding to the MREs in the LO promoter thus inhibited LO mRNA expression (Figure 4A). Note that upregulation of MT as a major phenotypic change was accompanied by downregulation of LO in CSC or Cd treated cells [40,76] and both MT and LO promoters contain MREs. Different phenotype changes in LO and MT genes in response to Cd may be explained by that many MRE-related transcription factors would be recruited to bind to MREs in the MT promoters for gene activation thus competing with their binding to the LO promoter. As reported, MT-I and MT-II genes each contain six MREs in their promoter regions, respectively, which display the different affinities for MTF-1 [67]. Apparently, LO MREs may have lower affinity than MT MREs for MTF-1 binding. In contrast to the LO MREs in response to Cd, the LO ARE is activated in Cd treated cells as evidenced by enhancement of Nrf2 binding to the LO ARE as examined by the ChIP assay (Figure 4B). Presumably, elevated ARE activities may play a critical role in maintaining the low basal level of the LO gene transcription under Cd stress conditions. NNK, a tobacco-specific carcinogen, is believed to contribute to the cancer burden in cigarette smokers [18]. Like Cd, NNK also inhibited activities of the LO MREs, but enhanced activities of the LO ARE (Figures 4A and 4B; reference [93]). NNK and Cd both have been shown to enhance methylation of CpG at the core promoter region of the LO gene (Figure 4E).

**Figure 5:**
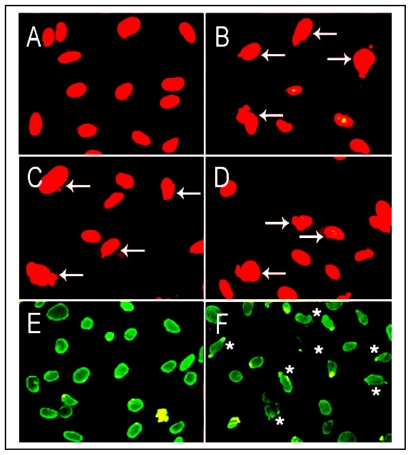
Alterations in nuclear organization in cells treated with CSC and BAPN, an inhibitor of LO. RFL6 cells were long-term incubated with graded concentrations of CSC from 80 to 160 μg/mL. Survival cells isolated with different degrees of CSC-resistance (CSCR) such as to 80, 120 and 160 μg/mL were referred to as CSCR80, CSCR120 and CSCR160. Arrows show altered nuclear morphology revealed by fluorescent DNA staining with propidium iodide. **A**, control; **B**, CSCR80; **C**, CSCR120; and **D**, CSCR160. Alterations in nuclear organization in RFL6 cells treated with BAPN, an LO inhibitor, at 100 μM for 7 days were revealed by histone H1 staining using the primary antibody against histone H1 and the secondary antibody conjugated with FITC. **E**, control; **F**, cells treated with BAPN. Asterisks show altered nuclear organization in BAPN-treated cells. Notably, both CSC and BAPN induced nuclear blebs.

Cytosine methylation in the CpG islands interferes with the interaction of DNA with proteins thus resulting in stable transcriptional repression [94,95]. Aberrant hypermethylation of genomic DNAs and increased levels of DNA methyltransferase (DNA MeTase) activity have been detected in NNK and Cd treated cells [22,23,96]. Furthermore, both NNK and Cd also suppressed histone H3 acetylation at the LO core promoter region (Figure 4C). The appropriate acetylation and deacetylation of H3 K56 is critical for chromatin assembly and genome stability [97]. Thus, inhibition of histone H3 acetylation is an important marker for inactivation of the LO gene by NNK and Cd. It should be noted that in addition to its action on histone H3, LO also oxidizes histone H1, a substrate of LO [42,43]. Thus, LO may be a key factor for the modification of nuclear structure proteins relevant to changes in chromatin configuration. Cells long-term treated with CSC or β-aminoproprionitrile (BAPN), that inhibits LO activity, exhibited an abnormal DNA organization manifested by appearance of nuclear blebs (Figure 5), the indices for chromosomal instability [98,99].

**Figure 6:**
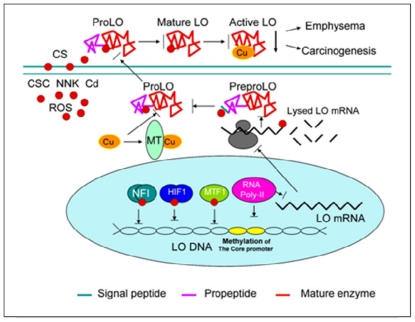
CS perturbs LO at transcriptional, translational and catalytic levels. At the promoter level, CS interferes with the interaction of redox-sensitive transcription factors such as NFI, HIF1, MTF-1 with corresponding *cis*-elements and induces methylation of CpG at the core promoter region inhibiting the initiation of LO gene transcription. At the mRNA levels, CS inhibition of both the synthesis and the stability of LO transcripts leads to decreased levels of steady-state LO mRNA. At the protein level, CS suppresses synthesis and processing of the preproLO such as signal peptide cleavage, and Cu binding to the proLO due to elevation of cellular thiols, resulting in reduction of the ProLO secretion to the ECM. At the catalytic levels mature apoLO without Cu cofactor binding exhibits the defect in enzyme activity. Thus, CS inhibition of the LO gene expression at multiple levels collectively contributes to decreased levels of the mature LO in the lung ECM favoring emphysema pathogenesis and carcinogenesis in the lung.

Thus, CSC, NNK and Cd, the major components of CS, perturbed LO at transcriptional, translational and catalytic levels representing a critical mechanism for CS toxicity to the lung (Figure 6).

## 5. Downregulation of LO and Pathogenesis of the Lung

### 5.1. LO Deficiency and Emphysema

Lung ECM, a dynamic structure, is composed of a number of functionally diverse elements which are integrated mainly by interstitial cells, e.g., fibroblasts [100,101]. The overall pattern of the lung ECM results from an intricate balance between the synthesis and degradation of its major structural components, e.g., collagen and elastin. Thus, the pathogenesis of emphysema, a lung ECM disease, is initiated by multiple mechanisms as revealed by animal model studies [7]. CS induced emphysema in humans [102]. It has been established that CS-enhanced degradation of the lung ECM proteins resulted from either the excess of protease production or the deficiency in α1-antitrypsin, an inhibitor of proteases, contributes to the onset of emphysema [7]. This review article aims at evaluation of the lung ECM protein synthesis and processing mechanism for emphysema pathology by emphasizing LO as a CS target. The critical role of LO in emphysema development was reflected in the disruption of the lung structure in chicks and rats following diet-induced deficiency of Cu, a cofactor for LO [78,79]. The lung lesions in these animals resembled panlobular emphysema in humans [80]. A mild form of emphysema was also produced in pigs raised on Cu deficient, Zn supplemented diet [103]. Zn, a known MT inducer, was used in this study based on its reported inhibitory effect on LO activity [104].

Patients with Menkes disease, an X-linked recessive disorder of Cu transport associated with the deficiency in LO, developed severe diffuse emphysema leading to respiratory failure and early death [105]. BAPN, an irreversible inhibitor of LO, can limit the fibrogenic response in certain surgical procedures and decrease collagen/elastin deposition in models of lung fibrosis. Feeding of BAPN markedly enhanced elastase-induced emphysema in hamsters [106]. Moreover, with simultaneous feeding of hamsters with BAPN, intratracheally instilled Cd resulted in lesions of emphysema but not fibrosis in the lung [107]. Additional evidence correlating LO deficiency with emphysema derives from studies of genetic variants of animals, e.g., blotchy mice with abnormal Cu transport, exhibited a deficiency of LO, inducing emphysema [108]. Furthermore, knockout mice deficient in LO, thus failed in crosslinking of collagen and elastin, resulted in major development problems in the lung manifested by emphysema [109]. Inactivation of LO and LO-like protein 1 individually caused enlarged airspaces of the lung [110,111]. It has long been known that cigarette smoke blocks cross-linking of elastin *in vitro* [112]. Our recent studies using CSC/NNK and Cd treated cells and Cd intratracheally instilled rats as *in vitro* and *in vivo* models have demonstrated downregulation of LO at multiple levels accompanied by inhibition of collagen and elastin expression in cell models and emphysema pathology in the lung of the animal model [40,76,88]. Modulation of the ECM gene expression by LO is supported by the observation that addition of BAPN, an inhibitor of LO, reduced mRNA levels of elastin in VSMC [113] and that overexpression of LO in COS-7 cells enhanced the promoter activity of the collagen type III gene [114].

### 5.2. LO Deficiency and Carcinogenesis in the Lung and Other Organs

Suppression of a set of tumor suppressor genes is a prerequisite to prosecute CS oncogenic programs in the lung. LO is a tumor suppressor gene as evidenced by its inhibitory effects on *ras* transformation activities [52]. Consistent with the anti-tumorigenic function of LO, normal rat kidney fibroblasts became transformed by expression of LO antisense displaying anchorage-independent growth and elevation of p21-*ras* expression [115]. Low levels of LO mRNA were detected in a variety of spontaneous human cancers [116] such as lung bronchogenic carcinoma [117], gastric cancers [118], head and neck squamous cell carcinoma [119], *etc.* Reduced LO mRNA expression was detected in various lung cancer cells, one of which (VRMC-LCD) exhibited complete methylation of the LO promoter and loss of LO expression which was reversed upon treatment with 5-aza-dC [120]. LO mRNA expressions in human bronchogenic carcinomas displayed a 3.4-fold graded reduction as tumors progressed from Stage I to IV, with a similar pattern of downregulation in LO proteins [117]. Moreover, microarray analysis demonstrated decreased LO mRNA expression in primary lung adenocarcinoma tissues and lung cancer cell lines compared to normal lung tissues and lung cells [120]. LO transcripts were progressively reduced in malignant prostate tumors either at primary or at metastatic lesions [121]. In breast tumors abundant amount of LO was observed in benign breast lesions surrounding *in situ* ductal carcinomas and in reactive fibrosis at the invasion front of infiltrating tumors. The level of LO was markedly decreased in late stromal reactions and nodetectable in the loose scirrhous stroma of invading ductal carcinomas [122,123], but increased in hypoxia-induced metastasis of breast cancers [51]. LO was absent in basal and squamous cell carcinoma and its knockout induced an invading phenotype in a skin equivalent model [124]. The LO gene was inactivated by methylation and loss of heterozygosity in human gastric cancers [118]. Reduction of LO expression as a result of somatic mutation of the LO gene was associated with human colorectal tumors [125]. Our recent studies have demonstrated that purified mature LO suppressed the proliferation of transformed cells by inactivation of growth factors such as bFGF [44]. Repression of *BCL*2, a proto-oncogene, by proLO and LO propeptide inhibited transformed phenotype of lung cancer cells (H1299) [120]. Moreover, LO modification of histone H1, H2 and H3, critical nuclear structural proteins, has been identified *in vitro* or in cells (Figure 4C, references [42,43,126]) and inhibition of LO enhanced chromatin instability (Figure 5). Therefore, modulating the expression of oncogenes such as *ras* and *BCL2*, regulating the activity of growth factors such as bFGF, and stabilizing nuclear proteins such as histone H1, H2 and H3 and thus maintaining chromatin and nuclear stabilities may be key mechanisms for LO tumor suppression. In view of novel functions of LO, downregulation of LO by CSC/NNK/Cd may severely impair its tumor suppressor properties triggering and facilitating carcinogenic programs in the lung.

## 6. Conclusions

In this paper, we have reviewed the literature for CS toxicity and our research findings, presented evidence for LO as a critical target for the insult of CSC, NNK and Cd, major components of CS, and emphasized the role of downregulation of LO, an enzyme with multiple biological functions, in the lung pathogenesis such emphysema and cancers. Further tracing CS effects on the interactions of LO with its substrates or binding ligands and exploring their underlying mechanisms are expected to provide the basis for prevention and treatment of CS-elicited lung diseases.
